# Angiopoietin-2 Serum Levels Improve Noninvasive Fibrosis Staging in Chronic Hepatitis C: A Fibrogenic-Angiogenic Link

**DOI:** 10.1371/journal.pone.0066143

**Published:** 2013-06-18

**Authors:** Ángel Hernández-Bartolomé, Rosario López-Rodríguez, Yolanda Rodríguez-Muñoz, Samuel Martín-Vílchez, María Jesús Borque, Luisa García-Buey, Leticia González-Moreno, Yolanda Real, Ricardo Moreno-Otero, Paloma Sanz-Cameno

**Affiliations:** 1 Liver Unit, Gastroenterology Service, Hospital Universitario de La Princesa, Instituto de Investigación Sanitaria Princesa, Universidad Autónoma de Madrid, and CIBERehd, Instituto de Salud Carlos III, Madrid, Spain; 2 Molecular Biology Unit, Hospital Universitario de La Princesa, Madrid, Spain; Institut Pasteur, France

## Abstract

**Aims:**

Accurate liver fibrosis staging is crucial for the management of chronic hepatitis C (CHC). The invasiveness and cost burden of liver biopsy have driven the search for new noninvasive biomarkers of fibrosis. Based on the link between serum angiopoietin-1 and 2 levels and CHC progression, we aimed to determine the value of these angiogenic factors as noninvasive biomarkers of liver fibrosis.

**Methods:**

Serum levels of angiopoietin-1 and -2 were measured by ELISA in 108 CHC patients who underwent pretreatment liver biopsy. The correlation between angiopoietins and clinical and demographic variables with liver fibrosis was analyzed by univariate regression. Significant factors were then subjected to multivariate analysis, from which we constructed a novel noninvasive liver fibrosis index (AngioScore), whose performance was validated in an independent series of 71 CHC patients. The accuracy of this model was compared with other documented fibrosis algorithms by De Long test.

**Results:**

Angiopoietins correlated significantly with hepatic fibrosis; however, only angiopoietin-2 was retained in the final model, which also included age, platelets, AST, INR, and GGT. The model was validated and behaved considerably better than other fibrosis indices in discriminating all, significant, moderate and severe liver fibrosis (0.886, 0.920, 0.923). Using clinically relevant cutoffs, we classified CHC patients by discarding significant fibrosis and diagnosing moderate and severe fibrosis with greater accuracy, sensitivity, and specificity.

**Conclusions:**

Our novel noninvasive liver fibrosis model, based on serum angiopoietin-2 levels, outperforms other indices and should help substantially in managing CHC and monitoring long-term follow-up prognosis.

## Introduction

Hepatitis C virus (HCV) infection is a major global health problem, because it effects chronic injury of the liver in a high percentage of acutely infected patients [1]. The persistence of inflammatory responses and cellular damage promote disease progression toward fibrosis, cirrhosis, and hepatocellular carcinoma [2], [3]. Thus, accurate evaluation of hepatic fibrosis has become the primary goal in managing the progression of chronic hepatitis C (CHC), because its morbidity and mortality are linked to cirrhosis and its complications.

In addition, the decision of physicians to administer antiviral therapy depends on the stage of fibrosis [4]. Liver biopsy (LB) is the standard for determining the stage of fibrosis, but due to its invasiveness, risk of complications, and potential for sampling errors, noninvasive markers are being sought as alternative diagnostic tools [5], [6], [7], [8], [9], [10].

Liver fibrosis results from an inappropriate wound-healing response to chronic damage that elicits excess deposition of matrix compounds and scarring, which ultimately alters liver structure and function [3]. Simultaneously, angiogenesis is stimulated to provide oxygen and nutrients to injured tissue; unfortunately, chronic damage dysregulates the repair processes, leading to capillarization of hepatic sinusoids, which restricts the blood supply, exacerbating tissue injury, fibrogenesis, and angiogenesis [11], [12], [13], [14]. This sequence of events appears to govern the progression of CHC to cirrhosis and hepatocellular carcinoma (HCC) [15], [16].

One of the most significant signaling pathways in pathological angiogenesis and HCC is the angiopoietin/Tie2 system [17], [18], [19]. Angiopoietin-1 and -2 (Ang1 and Ang2, respectively) are the most potent regulators of neovascularization, vascular remodelling, and maturation, through agonistic and antagonistic autophosphorylation, respectively, of their common tyrosine kinase receptor, Tie2 [20]. The balance between the signals that are generated by Ang1 and Ang2 has pathological effects–the Ang1/Ang2 equilibrium is altered in cancers, such as HCC, a highly vascularized tumor [21], [22], [23], and in diverse chronic liver diseases, such as primary biliary cirrhosis (PBC) and viral hepatitis B and C [24], [25], [26], [27], implicating angiopoietins as biomarkers of liver disease progression and putative therapeutic targets [28], [29].

We noted a link between serum Ang2 levels and the failure of response of CHC patients to antiviral treatment [25]; further, Ang2 and CHC progression have been associated directly in an earlier study [29]. Thus, we aimed to determine the value of angiopoietins in monitoring the evolution of CHC and preventing disease progression. This analysis resulted in the development of a novel noninvasive model of liver fibrosis (AngioScore), which was validated in an independent cohort of CHC patients, outperforming other documented procedures.

## Materials and Methods

### Patients

The training group included 108 CHC serum samples from the collection of the Liver Unit, Hospital Universitario La Princesa, randomly selected from patients without HIV, hepatitis B virus coinfection, or evidence of autoimmune liver disease who underwent pretreatment liver biopsy and gave written informed consent for their experimental use.

Similarly, the validation set comprised serum samples that were available in the same collection (total of 71) from CHC patients without coinfections or liver autoimmunity who underwent pretreatment liver biopsy and authorized their use for investigational purposes by signed informed consent.

The study protocol was approved on February 15, 2010 by the ethics committee of Hospital Universitario de La Princesa and was conducted per the Declaration of Helsinki.

Viral load in the serum of all patients was measured by COBAS AMPLICOR assay (Roche Molecular Systems, Branchburg, NJ), and HCV genotyping was performed by reverse-hybridization line probe assay (INNO-LiPAHCV; Innogenetics, Zwijndreht, Belgium). Serum samples were also subjected to routine laboratory tests at the biochemical laboratory of Hospital de La Princesa using common commercial methods. Total bilirubin, ALP, ALT, AST, GGT, albumin, serum cholesterol, prothrombin activity, INR, and platelets were measured.

### Liver Histology

Liver biopsies from patients were obtained by percutaneous needle extraction (Hepafix®, B. Braun Melsungen AG, Melsungen, Germany) under echographic control and were paraffin-embedded for routine anatomopathological examination. Histological features were analyzed in liver biopsies (median length 1.90 mm, 95% CI 1.78–2.02) per the METAVIR classification system [30], which scores the stage from F0 to F4: F0 is the absence of fibrosis, F1 is portal fibrosis without septa, F2 is portal fibrosis with rare septa, F3 is numerous septa without cirrhosis, and F4 is cirrhosis.

### Ang1 and Ang2 Concentrations in the Serum of CHC Patients

Circulating Ang1 and Ang2 levels were measured in the serum samples from CHC patients on the same day that they underwent liver biopsy–ie, before initiation of antiviral combination therapy–using the respective human ELISA kits following the manufacturer’s recommendations (Quantikine: R&D Systems, Minneapolis, MN). All measurements were made in duplicate, and the absorbance was read at 450 nm and corrected at 570 nm.

### Serological Indices of Fibrosis

Other liver fibrosis scores were calculated as follows:

1. APRI [31]: 100×(AST (IU/L)/40)/platelet count (10^9^/L)
2. FIB4 [32]: age (years)×AST (IU/L)/platelet count (10^9^/L)×ALT (IU/L)^½^
3. King [33]: age (years)×AST (IU/L)×INR/platelet count (10^9^/L)
4. AAR score [34]: AST (IU/L)/ALT (IU/L)
5. GUCI [35]: (AST×INR×100)/platelet count (10^9^/L)
6. LOK [36]: −5.56–(0.0089×platelet count (10^9^/L))+(1.26×AST (IU/L)/ALT (IU/L)) +5.27×INR
7. FORNS [37]: 7.811– (3.131×Ln platelet count (10^9^/L))+(0.781×Ln GGT (IU/L))+(3.467×Ln age (years)) –0.014×cholesterol
8. FI [38]: 8.0 − 0.01×platelet count (10^9^/L) − serum albumin (g/dL)
9. FCI [39]: (ALP×bilirubin)/(albumin×platelet count)

### Statistical Analysis

Comparisons of Ang1 and Ang2 serum levels between fibrosis stages were analyzed by Mann-Whitney U-test using a two-sided p-value; p<0.05 was considered significant. The relationship between angiopoietins and clinical and demographic variables with liver fibrosis was analyzed by univariate logistic regression. All variables that were significantly associated with fibrosis (p<0.05) were analyzed by step-backward multiple regression, in which none of the variables was forced to be included in the model. The covariates that were retained in the final model were used to build a novel liver fibrosis index (AngioScore), which was then validated with the validation set (n = 71).

The diagnostic performance of angiopoietins, the established fibrosis indices, and our novel fibrosis model was analyzed using area under the receiver operating characteristic curves (AUC-ROCs) to discriminate significant fibrosis (F>1), moderate fibrosis (F>2), and severe fibrosis (F>3). Sensitivity (Se), specificity (Sp), positive predictive value (PPV), negative predictive value (NPV), positive and negative likelihood ratio (LR), and accuracy (ACC) were calculated per standard methods and expressed as percentages. Optimal cutoff values were chosen to maximize the sum of sensitivity and specificity (Youden index) and were corrected according to the global prevalence of each fibrosis stage [40] (MedCalc version 12.3.0.0, Mariakerke, Belgium) to minimize the effects of selection bias. In addition, the clinical relevance of cutoffs that corresponded to sensitivities and specificities above 90% were examined.

The performance of AngioScore was compared with other fibrosis indices using the entire cohort of patients by De Long test (MedCalc 12.3.0.0, Mariakerke, Belgium).

## Results

### Demographics and Clinical Characteristics of CHC Patients

The main demographic, laboratory, and histological features of the training series of CHC patients (n = 108) were similar to those of the validation set (n = 71), except for AST and ALT, which were significantly higher in the training group (p<0.05 for both), and fibrosis stage–the training group included more patients with advanced fibrosis (p<0.005)–as shown in Table 1.

**Table 1:** Baseline characteristics of patients with chronic hepatitis C.

PATIENTS	T (n = 108)	V (n = 71)	p	ALL (n = 179)
**Sex (M/F)**	71/37	44/27	0.608	115/64
**Age (years)**	46 (25–65)	44(22–67)	0.275	45 (22–67)
**Viral load (×105 IU/mL)**	7.4 (3.0–13.0)	5.4 (2.2–12.0)	0.528	6.7 (2.2–13.0)
**HCV genotype, n (%)**			0.267	
1	84 (77.7)	60 (85.5)	–	144 (80.4)
Non-1	24 (22.3)	11 (15.5)	–	35 (19.6)
**Stage of liver fibrosis, n (%)**			**0.004**	
F1	25 (23.3)	17 (23.9)	–	42 (23.5)
F2	31 (28.7)	35 (49.6)	–	66 (36.9)
F3	33 (30.5)	7 (9.6)	–	40 (22.3)
F4	19 (17.5)	12 (16.9)	–	31 (17.3)
**AST (UI/L)**	56.0 (39.0–91.8)	46.0 (34.0–69.0)	**0.037**	53.0 (36.0–84.0)
**ALT (UI/L)**	88.5 (64.3–128.8)	69.0 (46.0–106.0)	**0.006**	81.0 (58.0–121.0)
**ALP (UI/L)**	123.5 (79.3–190.3)	135.0 (73.0–178.0)	0.667	126.0 (77.0–181.0)
**GGT (UI/L)**	48.5 (26.0–91.0)	36.0 (20.0–72.0)	0.066	45.0 (25.0–82.0)
**Prothrombin Activity (%)**	93.6 (85.5–101.0)	90.1(81.0–102.3)	0.428	92.8 (83.7–101.1)
**INR**	1.0 (0.9–1.1)	1.0 (0.9–1.1)	0.602	1.0 (0.9–1.1)
**Bilirrubin (mg/dL)**	0.7 (0.5–0.9)	0.7 (0.5–0.9)	0.965	0.7 (0.5–0.9)
**Platelet count (×109/L)a**	190.0 (151.0–224.0)	197.0 (160.0–237.0)	0.586	193.5 (152.0–232.5)
**Cholesterol Total (mg/dL)b**	171.5 (153.7–189.0)	165.0 (151.3–191.0)	0.367	169.0 (152.8–189.0)
**Albumin (g/dL)c**	4.3 (4.1–4.5)	4.4 (4.2–4.5)	0.374	4.3 (4.1–4.5)

Data are shown as number of patients (percentage) or median value (25th–75th percentile), except for age (median and range). ^a^ n = 178; ^b^ n = 174 and ^c^ n = 170. T, Training Group; V, Validation Group. Significant variables are shown in bold.

### Ang1 and Ang2 Serum Levels in the Training Cohort

**Figure 1:**
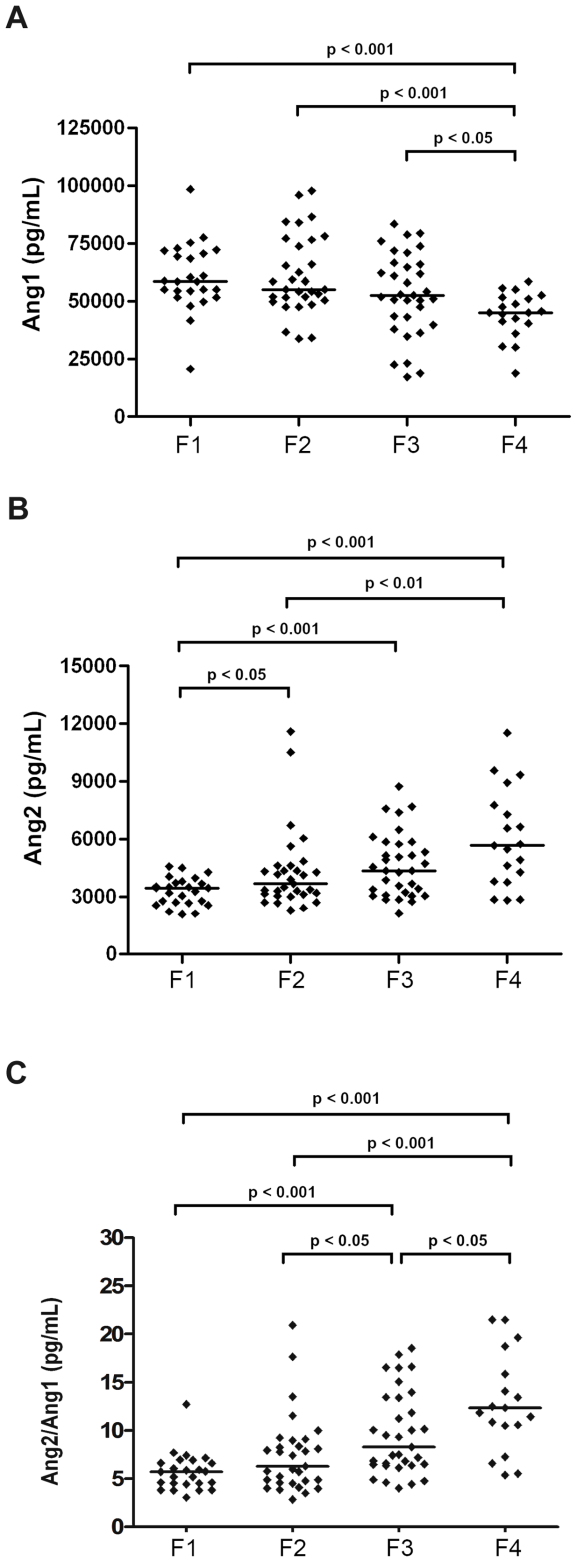
Serum levels of Ang1 and Ang2 in the training set of 108 CHC patients. Distribution of Angiopoietin-1 (A), Angiopoietin-2 (B), and Ang2/Ang1 (C) serum concentrations against METAVIR fibrosis stage. Medians are represented by horizontal lines. Two-sided p-values were calculated by non-parametric Mann-Whitney U Test.

Serum levels of Ang1 decreased gradually as the fibrosis progressed to more advanced stages–more so for F4 compared with lower stages (p<0.05, Figure 1a). In contrast, Ang2 serum concentrations rose progressively with the stage of fibrosis–a pattern that was significant between F1 and all other fibrosis stages and between F2 and F4 (Figure 1b). Notably, the Ang2/Ang1 ratio increased with the stage of fibrosis and differentiated F2 and F3, as well as F3 and F4 (p<0.05), the most clinically relevant endpoints (Figure 1c).

**Figure 2:**
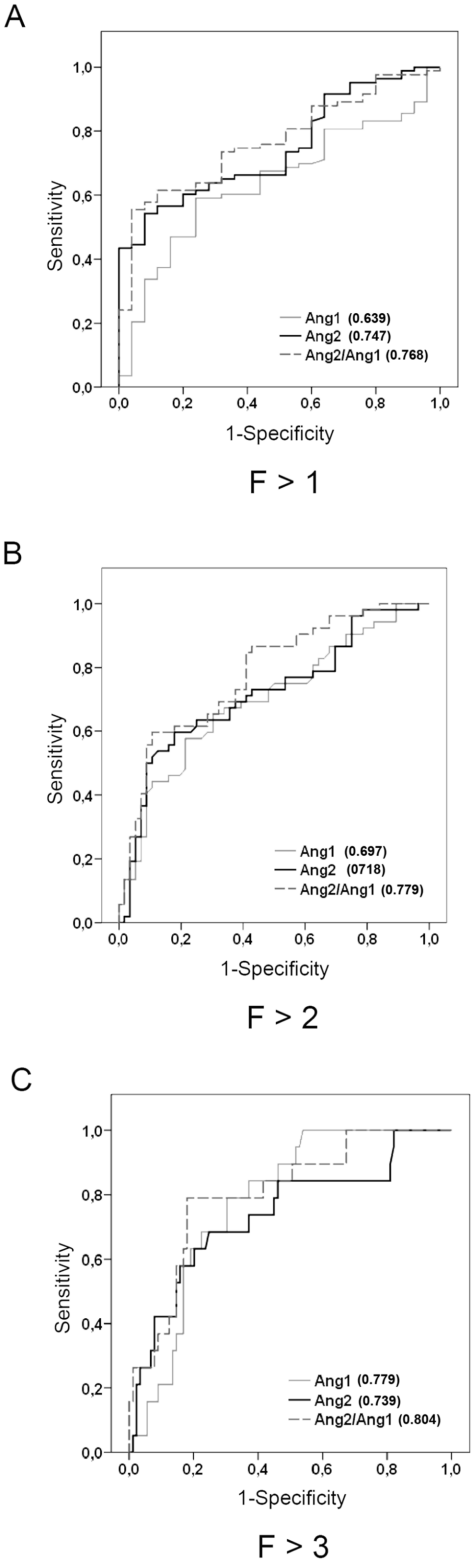
Performance of serum angiopoietin level in the training set of CHC patients. The area under the receiver operating characteristic curves (AUC-ROCs) of Ang1, Ang2, and Ang2/Ang1 for significant fibrosis (A), moderate fibrosis (B), and severe fibrosis-cirrhosis (C), respectively, are shown in brackets (n = 108).

By AUC-ROC analysis (Figure 2), Ang2 characterized patients with significant fibrosis stage (F>1), as did Ang1 in classifying cirrhotic individuals (AUC of 0.747 and 0.779, respectively), as suggested by Mann-Whitney U-test. Based on this contrast, we analyzed the predictive accuracy of the Ang2/Ang1 ratio and noted that it performed better than Ang1 and Ang2 alone, achieving an AUC of 0.804 for F>3.

### Liver Fibrosis-related Variables

The relationship between Ang1, Ang2, and demographic and clinical variables with liver fibrosis was first evaluated in the training set of CHC patients (n = 108). Serum Ang1 levels were inversely related to fibrosis (p = 0.01); in contrast, Ang2 was associated directly with fibrosis, as demonstrated by univariate regression analysis (p = 5.49×10^−6^). The Ang2/Ang1 ratio was linked to fibrosis, like AST, age, ALT, GGT, platelet count, INR, albumin, and bilirubin (Table 2).

**Table 2:** Association of angiopoietins and clinical and demographic variables with liver fibrosis in the training set of CHC patients.

Parameter	Standardized β Coefficient^a^	Nonstandardized Coefficient (95% CI)^a^	p value^a^	Standardized β Coefficient^b^	Nonstandardized coefficient (95%CI)^b^	p value^b^
**Ang1**	−0.310	3.352 (2.711–3.994)	**0.001**	0.020	1.20×10^−6^ (−8.17×10^−6^–1.05×10^−5^)	0.800
**Ang2**	0.422	2.1×10^−4^(1.2×10^−4^–3.0×10^−4^)	**5.49×10** ^−**6**^	0.167	8.14×10^−5^ (1.18×10^−5^–1.51×10^−4^)	**0.022**
**Ang2/Ang1**	0.417	0.062 (0.036–0.088)	**7.24×10** ^−**6**^	–	–	–
**Age**	0.344	0.038 (0.018–0.057)	**2.70×10** ^−**4**^	0.190	0.021 (0.006–0.036)	**0.006**
**AST**	0.435	0.009 (0.005–0.013)	**2.60×10** ^−**6**^	0.240	0.006 (0.002–0.009)	**0.004**
**ALT**	0.321	0.004 (0.002–0.006)	**0.001**	−0.095	−0.001 (−0.005–0.003)	0.504
**GGT**	0.351	0.004 (0.002–0.006)	**1.90×10** ^−**4**^	0.184	0.002 (3.02×10^−4^–0.004)	**0.021**
**ALP**	0.130	0.002 (−0.001–0.004)	0.180	–	–	–
**Platelet count^c^**	−0.518	−0.010 [−0.013–(−0.007)]	**1.08×10** ^−**8**^	−0.271	−0.005 [−0.008–(−0.002)]	**0.000**
**INR**	0.452	4.916 (3.048–6.783)	**9.06×10** ^−**7**^	0.323	3.369 (1.855–4.882)	**0.000**
**Albumin^d^**	−0.205	−0.594 [−1.160–(−0.029)]	**0.039**	0.062	0.181 (−0.246–0.608)	0.402
**Total Cholesterol^e^**	−0.109	−0.003 (−0.009–0.003)	0.268	–	–	–
**Total Bilirubin**	0.205	0.651 (0.051–1.250)	**0.034**	0.021	0.065 (−0.384–0.513)	0.775

a Univariate regression p value; ^b^Multivariate regression p value; n = 108, except for ^c^n = 107, ^d^n = 101, ^e^n = 106. Significant variables are shown in bold.

All variables that were significantly related to liver fibrosis (Ang1, Ang2, Ang2/Ang1, age, AST, ALT, GGT, platelet count, INR, albumin, and bilirubin) were analyzed together by step-backward multiple regression, wherein Ang2, age, platelet count, INR, AST, and GGT were associated independently with liver fibrosis (Table 2). However, Ang1, ALT, albumin, and bilirubin were not effective when analyzed in combination with the other covariates and were automatically removed from the multivariate model.

### Validation and Performance of a New Index for Noninvasive Assessment of Liver Fibrosis

The novel liver fibrosis model was applied to the training group of CHC patients (n = 107, because platelet count was unavailable in 1 patient) and was accurate in discerning all, F>1, F>2, and F>3 fibrosis (AUC >0.9). The model continued to perform well when tested with the validation set (n = 71), discriminating F>2 (AUC >0.8) and F>3 (AUC >0.9), the chief clinical endpoints (Table S1).

Next, the fibrosis-related variables were included in AngioScore, a new noninvasive model of CHC liver fibrosis that was calculated using the total population (n = 178), after considering their distribution functions, as follows:
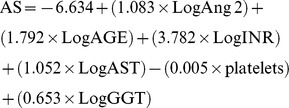



This model was then compared with other fibrosis indices (APRI, FIB4, King, AAR, GUCI, Lok, Forns, FI, and FCI) for the 178 CHC patients; their correlation with liver fibrosis stage, reflected that our model performed best (p<0.0001, Table S2).

In addition, the diagnostic performance of all indices in the entire cohort was evaluated by AUC-ROC analysis, which indicated that our model was superior to the others in staging liver fibrosis: AUC values of 0.886 for F>1, 0.920 for F>2, and 0.923 for F>3. The cutoffs that corresponded to the Youden index for our model had a sensitivity and specificity of approximately 80% or higher and accuracy above 80% for all stages (F>1, F>2, and F>3; Table 3).

**Table 3:** Accuracy of AngioScore and other liver fibrosis indices in the total cohort of CHC patients.

F>1															
	AUC-ROC (95%CI)	Cutoff^a^	Se	95%CI	Sp	95%CI	+LR	95%CI	−LR	95%CI	+PV	95%CI	−PV	95%CI	ACC
**AS**	0.886 (0.829–0.928)	>1.9248	79.41	71.6–85.9	83.33	68.6–93.0	4.76	2.4–9.4	0.25	0.200–0.400	81.5	71.4–89.2	81.4	72.1–88.7	81.5
**APRI**	0.822 (0.758–0.875)	>0.7158	58.09	49.3–66.5	90.48	77.4–97.3	6.10	2.4–15.7	0.46	0.400–0.600	84.9	73.1–93.0	70.0	61.0–78.1	74.9
**FIB4**	0.855 (0.795–0.903)	>1.2030	72.06	63.7–79.4	83.33	68.6–93.0	4.32	2.2–8.6	0.34	0.200–0.500	80.0	69.2–88.3	76.4	66.9–84.3	77.9
**KING**	0.855 (0.795–0.903)	>10.6470	72.06	63.7–79.4	85.71	71.5–94.6	5.04	2.4–10.7	0.33	0.200–0.400	82.3	71.7–90.2	76.9	67.5–84.6	79.2
**AAR**	0.554 (0.447–0.628)	>0.6486	54.01	45.3–62.6	61.90	45.6–76.4	1.42	0.9–2.1	0.74	0.600–1.000	56.7	45.2–67.7	59.3	48.9–69.2	58.1
**GUCI**	0.816 (0.751–0.870)	>30.4200	57.35	48.6–65.8	92.86	80.5–98.5	8.03	2.7–24.1	0.46	0.400–0.600	88.1	76.5–95.3	70.2	61.3–78.2	75.8
**LOK**	0.683 (0.609–0.750)	>–1.5009	82.35	74.9–88.4	42.86	27.7–59.0	1.44	1.1–1.9	0.41	0.200–0.700	57.1	47.9–66.0	72.5	58.6–83.7	61.8
**FORNS^b^**	0.860 (0.799–0.908)	>4.5887	78.03	70.0–84.8	82.93	67.9–92.8	4.57	2.3–9.0	0.26	0.200––0.400	80.8	70.5–88.8	80.4	70.8–87.9	80.6
**FI^c^**	0.675 (0.598–0.745)	>1.8300	48.46	39.6–57.4	89.74	75.8–97.1	4.73	1.8–12.2	0.57	0.500–0.700	81.3	67.5–91.1	65.4	56.1–73.8	69.9
**FCI^d^**	0.522 (0.443–0.599)	>0.1943	20.00	13.5–27.9	94.74	82.3–99.4	3.80	0.9–15.3	0.84	0.800–0.900	77.8	53.9–93.0	56.2	47.8–64.4	58.9
**F>2**															
	**AUC-ROC (95%CI)**	**Cutoff** a	**Se**	**95%CI**	**Sp**	**95%CI**	**+LR**	**95%CI**	**−LR**	**95%CI**	**+PV**	**95%CI**	**−PV**	**95%CI**	**ACC**
**AS**	0.920 (0.870–0.955)	>2.3967	81.43	70.3–89.7	91.67	84.8–96.1	9.77	5.2–18.4	0.2	0.100–0.300	77.4	63.1–88.2	93.4	87.6–97.0	89.0
**APRI**	0.882 (0.825–0.925)	>0.8069	80.00	68.7–88.6	86.11	78.1–92.0	5.76	3.5–9.3	0.23	0.100–0.400	66.9	52.9–79.0	92.5	86.2–96.5	84.5
**FIB4**	0.880 (0.823–0.924)	>1.7538	72.86	60.9–82.8	87.04	79.2–92.7	5.62	3.4–9.4	0.31	0.200–0.500	66.4	51.6–79.1	90.1	83.6–94.7	83.4
**KING**	0.896 (0.842–0.937)	>12.8571	87.14	77.0–93.9	75.93	66.7–83.6	3.62	2.6–5.1	0.17	0.090–0.300	56.0	43.8–67.7	94.4	88.1–97.9	78.8
**AAR**	0.617 (0.542–0.689)	>0.7363	45.07	33.2–57.3	77.78	68.8–85.2	2.03	1.3–3.1	0.71	0.600–0.900	41.6	27.8–56.4	80.1	72.1––86.6	69.3
**GUCI**	0.881 (0.825–0.925)	>32.1370	82.86	72.0–90.8	82.41	73.9–89.1	4.71	3.1–7.2	0.21	0.100–0.400	62.3	49.0–74.4	93.2	87.0–97.0	82.5
**LOK**	0.771 (0.702–0.831)	>–0.7447	70.00	57.9–80.4	75.00	65.7–82.8	2.80	2.0–4.0	0.40	0.300–0.600	49.6	36.9–62.3	87.7	80.1–93.1	73.7
**FORNS^b^**	0.857 (0.796–0.905)	>5.0262	91.18	81.8–96.7	68.57	58.8–77.3	2.90	2.2–3.9	0.13	0.060–0.300	50.5	39.1–61.8	95.7	89.2–98.8	74.4
**FI^c^**	0.764 (0.693–0.826)	>1.8300	70.77	58.2–81.4	79.81	70.8–87.0	3.5	2.3–5.3	0.37	0.200–0.500	55.2	41.3–68.5	88.6	81.2–93.8	77.5
**FCI^d^**	0.696 (0.621–0.765)	>0.1513	47.69	35.1–60.5	84.47	76.0–90.9	3.07	1.8–5.2	0.62	0.500–0.800	51.9	35.6–67.9	82.1	74.3–88.4	74.9
**F>3**															
	**AUC**–**ROC (95%CI)**	**Cutoff** a	**Se**	**95%CI**	**Sp**	**95%CI**	**+LR**	**95%CI**	–**LR**	**95%CI**	**+PV**	**95%CI**	–**PV**	**95%CI**	**ACC**
**AS**	0.923 (0.873–0.957)	>2.5393	96.77	83.3–99.9	78.91	71.4–85.2	4.59	3.3–6.3	0.041	0.006–0.300	38.5	25.5–52.9	99.4	96.0–100.0	81.1
**APRI**	0.887 (0.831–0.930)	>0.7401	100.00	88.8–100	67.35	59.1–74.8	3.06	2.4–3.9	0	–	29.5	19.3–41.4	100.0	96.5–100.0	71.3
**FIB4**	0.858 (0.978–0.906)	>1.5558	93.55	78.6–99.2	70.07	62.0–77.3	3.13	2.4–4.1	0.092	0.020–0.400	29.9	19.2–42.4	98.8	94.5–99.9	72.9
**KING**	0.878 (0.821–0.923)	>16.6327	90.32	74.2–98.0	73.47	65.6–80.4	3.40	2.5–4.6	0.130	0.040–0.400	31.7	20.3–45.0	98.2	93.9–99.8	75.5
**AAR**	0.643 (0.568–0.713)	>0.5444	96.77	83.3–99.9	31.08	23.7–39.2	1.40	1.2–1.6	0.100	0.010–0.700	16.1	10.2–23.6	98.6	90.2–100.0	39.0
**GUCI**	0.896 (0.841–0.936)	>32.9412	100.00	88.8–100.0	70.07	62.0–77.3	3.34	2.6–4.3	0	–	31.3	20.6–43.7	100.0	96.7–100.0	73.7
**LOK**	0.882 (0.825–0.926)	>–0.3791	83.87	66.3–94.5	85.71	79.0–90.9	5.87	3.8–9.0	0.19	0.080–0.400	44.5	28.8–61.0	97.5	93.3–99.4	85.5
**FORNS^b^**	0.849 (0.787–0.899)	>6.5692	70.00	50.6–85.3	85.31	78.4–90.7	4.77	3.0–7.5	0.35	0.200–0.600	39.4	23.6–57.0	95.4	90.4–98.3	83.5
**FI^c^**	0.805 (0.737–0.861)	>2.0300	75.86	56.5–89.7	79.29	71.6–85.7	3.66	2.5–5.4	0.30	0.200–0.600	33.3	20.1–48.7	96.0	90.8–98.7	78.9
**FCI^d^**	0.750 (0.677–0.813)	>0.2069	48.28	29.4–67.5	92.81	87.2–96.5	6.71	3.3–13.6	0.56	0.400–0.800	47.8	25.3–70.9	92.9	87.5–96.5	87.5

a Youden index criterion; Se, Sensitivity; Sp, Specificity; +LR, Positive Likelihood ratio; -LR, Negative Likelihood ratio; +PV, Positive predictive value; -PV, Negative predictive value; ACC, Accuracy. n = 178 CHC patients except for ^b^n = 173, ^c^n = 169 and ^d^n = 168.

Moreover, the precision of our index was significantly better than most fibrosis scores in differentiating all, significant, moderate, and severe fibrosis (De Long test, p<0.05; Table 4).

**Table 4:** Comparison of AUC values from all noninvasive liver fibrosis indices in the total cohort of CHC patients.

F>1			
INDICES	AUC-ROC (95% CI)	Standard Error	p
**AS**	0.886 (0.829–0.928)	0.0273	–
**APRI**	0.822 (0.758–0.875)	0.0359	**0.012**
**FIB4**	0.855 (0.795–0.903)	0.0312	0.272
**KING**	0.855 (0.795–0.903)	0.0315	0.132
**ASTALT**	0.554 (0.477–0.628)	0.0533	**<0.001**
**GUCI**	0.816 (0.751–0.870)	0.0362	**0.004**
**LOK**	0.683 (0.609–0.750)	0.0478	**<0.001**
**FORNS^a^**	0.860 (0.799–0.908)	0.0322	0.245
**FI^b^**	0.675 (0.598–0.745)	0.0456	**<0.001**
**FCI^c^**	0.522 (0.443–0.599)	0.0513	**<0.001**
**F>2**			
**INDICES**	**AUC**-**ROC (95% CI)**	**Standard Error**	**p**
**AS**	0.920 (0.870–0.955)	0.0204	–
**APRI**	0.882 (0.825–0.925)	0.0260	**0.047**
**FIB4**	0.880 (0.823–0.924)	0.0249	0.060
**KING**	0.896(0.842–0.937)	0.0229	0.121
**ASTALT**	0.617 (0.542–0.689)	0.0435	**<0.001**
**GUCI**	0.881 (0.825–0.925)	0.0260	**0.035**
**LOK**	0.771 (0.702–0.831)	0.0356	**<0.001**
**FORNS^a^**	0.857 (0.796–0.905)	0.0277	**0.003**
**FI^b^**	0.764 (0.693–0.826)	0.0392	**<0.001**
**FCI^c^**	0.696 (0.621–0.765)	0.0424	**<0.001**
**F>3**			
**INDICES**	**AUC-ROC (95% CI)**	**Standard Error**	**p**
**AS**	0.923 (0.873–0.957)	0.0217	–
**APRI**	0.887 (0.831–0.930)	0.0254	0.084
**FIB4**	0.858 (0.798–0.906)	0.0316	**0.011**
**KING**	0.878 (0.821–0.923)	0.0269	**0.027**
**ASTALT**	0.643 (0.568–0.713)	0.0503	**<0.001**
**GUCI**	0.896 (0.841–0.936)	0.0244	0.162
**LOK**	0.882 (0.825–0.926)	0.0328	0.179
**FORNS^a^**	0.849 (0.787–0.899)	0.0335	**0.004**
**FI^b^**	0.805 (0.737–0.861)	0.0476	**0.001**
**FCI^c^**	0.750 (0.677–0.813)	0.0538	**<0.001**

Two sided p values by De Long test. n = 178, except for ^a^n = 173, ^b^n = 169 and ^c^n = 168. AS, AngioScore.

Because the distribution of fibrosis stages in our population differed from that of a large population (33,121 CHC patients [40], yielding 48% vs 72% for F>1, 26% vs 40% for F>2, and 12% vs 18% for F>3, respectively), the optimal cutoffs for all liver fibrosis indices were recalculated accordingly (MedCalc version 12.3.0.0). This analysis yielded the same optimal diagnostic parameters for our liver fibrosis model, except for 1 discrepancy–the optimal cutoff for F>3 was 3.0687 instead of 2.5393, obtained by the Youden index, demonstrating high specificity (93.88%) and acceptable sensitivity (67.74%). Once the corrected optimal cutoff for F>3 was applied, the accuracy of our model reached 90.7% (Table S3).

### Diagnostic Criteria

**Figure 3:**
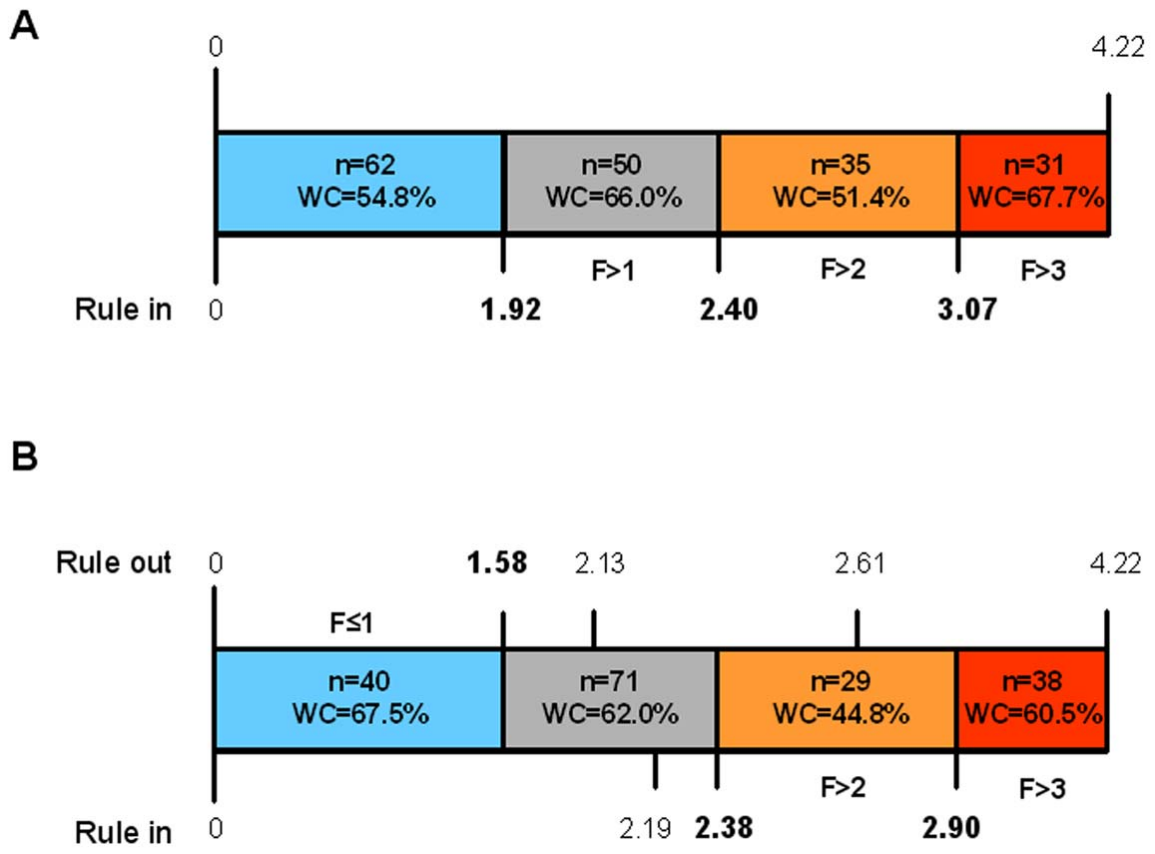
Clinically relevant criteria of a novel noninvasive liver fibrosis index, AngioScore. Diagnostic criteria of AngioScore obtained from the total cohort of CHC patients (n = 178). A) Corrected optimal cutoffs for diagnosing F>1, F>2, and F>3. B) Cutoffs obtained for sensitivities and specificities above 90%. Criteria for excluding F>1 and identifying F>2 and F>3 are shown in bold. n: number of patients in the corresponding category; WC: well-classified patients.

Based on the clinical relevance of accurately predicting CHC patients without significant fibrosis (F≤1) and those with moderate or advanced fibrosis, we examined the cutoffs that corresponded to sensitivities or specificities over 90% in addition to the optimal cutoffs (Figure 3). A value of 1.58, corresponding to a specificity of above 90%, identified patients with low fibrosis, correctly classifying 67.5% of patients with an accuracy of 76.8% (Figure 3); however, the optimal cutoffs were more effective in identifying patients with moderate and severe fibrosis (51.4% and 67.7% of well-classified patients, respectively) than those related to specificities >90%–2.40 *vs* 2.38 and 3.07 *vs* 2.90, respectively, with sensitivities of 81.43% and 74.19% and comparable accuracies (88.3% and 88.5%).

An in-depth examination of all diagnostic criteria for all liver fibrosis indices (Youden, optimal, above 90% sensitivity, and over 90% specificity) demonstrated the superior accuracy of our model in distinguishing all, significant, moderate, and severe liver fibrosis (Table S3). Only the optimal cutoffs for GUCI and FCI had greater accuracy for F>3 than that of the novel index (91.06 and 91.72 *vs.* 90.74, respectively) but had worse sensitivity (35.48 and 31.03 *vs.* 67.74, respectively).

## Discussion

Chronic liver damage (CLD) often leads to advanced fibrosis and cirrhosis, due to the persistent and altered stimulation of immune responses and tissue repair mechanisms. Excess deposition of extracellular matrix (ECM) components distorts the hepatic architecture by forming ECM complexes and fibrous scars. Fibrosis is a fundamental complication of CLD, which often progresses to cirrhosis and HCC after 15 to 20 years in a significant proportion of HCV-infected patients [2], [41].

Liver biopsy is considered the standard method of diagnosing and staging liver disease, based on its value in assessing the stage of fibrosis and necroinflammatory grade [42], [43]. However, this procedure is imprecise (yielding equivocal results in one-third of cases) and has serious limitations–it is highly invasive and expensive and can cause major complications. Further, fibrosis is a marker rather than the actual endpoint of CLD progression; thus, LB is not reasonable for the repetitive assessment of liver fibrosis during the long-term follow-up of patients, necessitating noninvasive markers that accurately screen the progression of CLD before, during, and after treatment [6], [44].

Several scores that are based on routine laboratory tests and more direct fibrosis biomarkers have been proposed to assess CHC progression, such as the aspartate aminotransferase-to-platelet ratio index [34], Forns index [37], Fibrometer (BBL Fibro System) [45], FibroSpect [46], Hepascore [47], Fibrotest (Biopredictive) [48], and MP3 [49] score–of which Fibrotest is the most validated and has recently been recommended in France as the first-line method for assessing CHC fibrosis.

Measurement of liver stiffness by transient elastography–Fibroscan (Echosens, Paris) [50]–is another noninvasive method that is approved for evaluating liver fibrosis and is useful for verifying cirrhosis (AUC>0.90), but it is notably less reliable in diagnosing significant fibrosis (AUC<0.80). Moreover, Fibroscan has several limitations, such as equipment cost, the need for extensive operator experience, and the existence of confounding factors, such as obesity and inflammation.

Based on these findings, the use of noninvasive methods is increasingly being recommended for liver fibrosis staging, but current tools are insufficient in supplanting LB; thus, they are more likely to aid a diagnosis when combined in algorithms, reserving LB for cases in which the accuracy of noninvasive tests is inadequate–avoiding a significant number of biopsies from being performed [6], [7], [51].

Notably, intrahepatic pathological angiogenesis is observed frequently during CLD and is linked to fibrogenesis [14]. The progression to cirrhosis correlates with enhanced vascular density and the expression of angiogenic factors, such as VEGF-A, Ang1, and Ang2, in cirrhotic rats [52], [53], and studies have suggested that liver pathologies, such as focal nodular hyperplasia, cirrhosis, adenomas, and HCC, have a common etiology, for which Ang-1 and Ang-2 are the most informative markers for diagnosis. Similarly, serum Ang-2 levels are elevated in patients with cirrhosis of various etiologies, suggesting that Ang-2 is a marker that can be used to detect the degree of liver injury [24], [26], [28], [54].

Our study, initially performed in 108 CHC patients and validated in an independent cohort of 71 CHC patients, demonstrates the value of Ang2 as a fibrosis marker, confirming earlier findings [25], [28], [29]. Ang1 and Ang2 levels correlated well but inversely with fibrosis stage and performed adequately, particularly Ang-2, which had AUC- values above 0.70 in discriminating all, significant, moderate, and severe fibrosis. The involvement of angiopoietin/Tie2 signaling in vascular homeostasis, immune regulation, and tissue remodeling–processes that are significantly altered in CLD–highlights the importance of these data. Further, elevated Ang2 expression levels have been linked to cancer, particularly HCC; thus, the value of this factor in monitoring CLD progression might surpass its significance as a tool in assessing fibrosis.

Our model, which comprises all independent significant variables–age, platelet count, INR, AST, and GGT, which have been linked to liver fibrosis [10]–and Ang2, was accurate in discriminating F>1, F>2, and F>3 (AUC >0.910, all) in the training set of CHC patients (n = 107). Notably, this model retained its outstanding precision with the validation group and had greater accuracy and correlation with fibrosis than other noninvasive fibrosis indices–APRI, FIB4, KING, and AAR, GUCI, Lok, Forns, FI, FCI–in the total cohort of CHC patients (Table 4 and Table S2).

The AUC values of AngioScore were the highest with regard to the diagnosis of F>1 (0.886), F>2 (0.920), and F>3 (0.923), suggesting that it is an effective test that performs accurately in discriminating liver fibrosis stages, minimizing possible selection bias that is attributable to prevalence. Nonetheless, when the data were corrected and analyzed by fibrosis prevalence from a documented extensive cohort of CHC patients (n = 33,121) [40], the liver fibrosis model had the same or better accuracy. Further, the excellent AUC values of our model in discriminating between fibrosis stages (around 0.9) demonstrates that it functions as well as LB [42].

The analysis of the criteria of our model yielded a diagnostic cutoff 1.58 in discarding significant fibrosis with elevated sensitivity (>90%) and well-classified patients (67.5%) and demonstrated the significance of 2.40 and 3.07 as optimal values of this index in diagnosing moderate and severe fibrosis, respectively, with high specificity (>90%) and accuracy (∼90%); by applying these criteria, 83.4% and 67.7% of patients were identified as having F>2 and F>3, respectively. Although the optimal values for the GUCI and FCI indices had marginally better accuracy (91.1% and 91.7%, respectively) than our model (90.7%) in discriminating F>3 (Table 3), they had weaker sensitivity (35.48% and 31.03% *vs.* 67.64%, respectively).

Unfortunately, the high cost of Fibrotest prevented us from comparing it with our novel liver fibrosis model; however, AngioScore has shown better diagnostic accuracy for all significant, moderate, and severe fibrosis than Fibrotest and Fibroscan–the most widely used noninvasive methods of liver fibrosis staging–in most studies [55].

Although our findings validate Ang2 as a valuable biomarker of liver fibrosis, they are preliminary; thus, further studies in extended cohorts of patients should be performed to verify the significance of our model in monitoring the evolution of CHC and its potential to predict the progression of other CLDs. In addition, this model is less complicated and cheaper, because it can be calculated easily from standard parameters (age, platelet count, INR, AST, and GGT) and serum Ang2 levels during a patient’s visit, obviating the need for complex formulas and costly equipment. Moreover, this model significantly improves the precision of other indices of liver fibrosis. Our index is more accurate, less invasive, and cheaper than many existing predictive tools for liver fibrosis staging and can help clinical decision-making during patient follow-up.

In conclusion, angiopoietins correlate significantly with liver fibrosis, and our new fibrosis model–comprising Ang2, age, platelet count, INR, AST, and GGT–predicts significant, moderate and severe fibrosis-cirrhosis in CHC patients with outstanding accuracy and superior diagnostic performance compared with other noninvasive liver fibrosis indices. Further studies should confirm its efficacy for CHC and CLDs of distinct etiologies.

## Supporting Information

Table S1 **Comparisons between AngioScore AUC-ROCs from training and validation groups of CHC patients.**
(DOC)

Table S2 **Correlations of different liver fibrosis indices with liver fibrosis in the total cohort of CHC patients.**
(DOC)

Table S3 **Comparisons among different diagnostic criteria from analyzed liver fibrosis indices fibrosis in the total studied cohort of CHC patients.**
(DOC)
