# Supplementary material for: Angiopoietin-2 Serum Levels Improve Noninvasive Fibrosis Staging in Chronic Hepatitis C: A Fibrogenic-Angiogenic Link
Source: PLoS One. 2013 Jun 18;8(6):e66143. doi: 10.1371/journal.pone.0066143 (PMC3688858; doi:10.1371/journal.pone.0066143)
Supplement: Table S1 — Comparisons between AngioScore AUC-ROCs from training and validation groups of CHC patients. (DOC) [file pone.0066143.s001.doc]

**Table S1.** Comparisons between AngioScore AUC-ROCs from training and validation groups of CHC patients

| **CHC Group** | **AS AUC-ROC (95% CI)** | **Standard Error** | **p** |
| --- | --- | --- | --- |
| **F>1** | | | |
| **Training** | 0.940 (0.877-0.977) | 0.024 | - |
| **Validation** | 0.753 (0.629-0.876) | 0.063 | 0.005 |
| **F>2** | | | |
| **Training** | 0.941 (0.878-0.977) | 0.022 | - |
| **Validation** | 0.831 (0.705-0.957) | 0.064 | 0.100 |
| **F>3** | | | |
| **Training** | 0.918 (0.849-0.962) | 0.026 | - |
| **Validation** | 0.931 (0.842-1.019) | 0.045 | 0.800 |

AS, AngioScore model for liver fibrosis staging; CI, confidence interval; Training group (n=107); V, validation group (n=71). Two sided p values of training vs validation groups by De Long test.
