# Supplementary material for: Angiopoietin-2 Serum Levels Improve Noninvasive Fibrosis Staging in Chronic Hepatitis C: A Fibrogenic-Angiogenic Link
Source: PLoS One. 2013 Jun 18;8(6):e66143. doi: 10.1371/journal.pone.0066143 (PMC3688858; doi:10.1371/journal.pone.0066143)
Supplement: Table S2 — Correlations of different liver fibrosis indices with liver fibrosis in the total cohort of CHC patients. (DOC) [file pone.0066143.s002.doc]

**Table S2.** Correlations of different liver fibrosis indices with liver fibrosis in the total cohort of CHC patients.

| **Index** | **Standardized** **β Coeffcient** | **Nonstandardized B Coefficient** **(95% CI)** | **p value^a^** |
| --- | --- | --- | --- |
| **AS** | 0.779 | 1.000 (0.880-1.120) | 1.68X10^-37^ |
| **APRI** | 0.596 | 0.733 (0.586-0.880) | 1.72x10^-18^ |
| **FIB4** | 0.592 | 0.446 (0.355-0.536) | 7.24x10^-18^ |
| **KING** | 0.590 | 0.030 (0.024-0.036) | 4.70x10^-18^ |
| **AAR** | 0.199 | 0.895 (0.241-1.548) | 0.008 |
| **GUCI** | 0.612 | 0.017 (0.014-0.020) | 1.16x10^-19^ |
| **LOK** | 0.527 | 0.608 (0.462-0.754) | 4.25x10^-14^ |
| **FORNS** | 0.667 | 0.392 (0.326-0.458) | 1.27x10^-23^ |
| **FI** | 0.469 | 0.661 (0.470-0.851) | 1.29x10^-10^ |
| **FCI** | 0.344 | 1.429 (0.831-2.026) | 4.96x10^-6^ |

AS, AngioScore.  ^a^Univariate regression p value.
