# Supplementary material for: Angiopoietin-2 Serum Levels Improve Noninvasive Fibrosis Staging in Chronic Hepatitis C: A Fibrogenic-Angiogenic Link
Source: PLoS One. 2013 Jun 18;8(6):e66143. doi: 10.1371/journal.pone.0066143 (PMC3688858; doi:10.1371/journal.pone.0066143)
Supplement: Table S3 — Comparisons among different diagnostic criteria from analyzed liver fibrosis indices fibrosis in the total studied cohort of CHC patients. (DOC) [file pone.0066143.s003.doc]

**Table S3. Comparisons among different diagnostic criteria from analyzed liver fibrosis indices fibrosis in the total studied cohort of CHC patients.**

| **F>1** | | | | | | | | | | | | | | | |
| --- | --- | --- | --- | --- | --- | --- | --- | --- | --- | --- | --- | --- | --- | --- | --- |
|  | **AUC** | **Cutoff** | **Se(%)** | **CI** | **Sp(%)** | **CI** | **+LR(%)** | **CI** | **-LR(%)** | **CI** | **+PV(%)** | **CI** | **-PV(%)** | **CI** | **ACC(%)** |
| **AS^1^** | 0.886 (0.829-0.928) | >1.9248 | 79.41 | 71.6-85.9 | 83.33 | 68.6-93.0 | 4.76 | 2.4-9.4 | 0.25 | 0.20-0.40 | 81.5 | 71.4-89.2 | 81.4 | 72.1-88.7 | **81.5** |
| **AS^2^** | 0.886 (0.829-0.928) | >1.9248 | 79.41 | 71.6-85.9 | 83.33 | 68.6-93.0 | 4.76 | 2.4-9.4 | 0.25 | 0.20-0.40 | 81.5 | 71.4-89.2 | 81.4 | 72.1-88.7 | **81.5** |
| **AS^3^** | 0.886 (0.829-0.928) | >1.5848 | 90.44 | 84.2-94.8 | 64.29 | 48.0-78.4 | 2.53 | 2.0-3.2 | 0.15 | 0.08-0.30 | 70.0 | 60.6-78.4 | 87.9 | 77.7-94.6 | **76.8** |
| **AS^4^** | 0.886 (0.829-0.928) | >2.1906 | 61.03 | 52.3-69.3 | 90.48 | 77.4-97.3 | 6.41 | 2.5-16.4 | 0.43 | 0.30-0.50 | 85.5 | 74.1-93.3 | 71.6 | 62.5-79.5 | **76.3** |
| **APRI^1^** | 0.822 (0.758-0.875) | >0.7158 | 58.09 | 49.3-66.5 | 90.48 | 77.4-97.3 | 6.10 | 2.4-15.7 | 0.46 | 0.40-0.60 | 84.9 | 73.1-93.0 | 70.0 | 61.0-78.1 | **74.9** |
| **APRI^2^** | 0.822 (0.758-0.875) | >0.7158 | 58.09 | 49.3-66.5 | 90.48 | 77.4-97.3 | 6.10 | 2.4-15.7 | 0.46 | 0.40-0.60 | 84.9 | 73.1-93.0 | 70.0 | 61.0-78.1 | **74.9** |
| **APRI^3^** | 0.822 (0.758-0.875) | >0.3938 | 90.44 | 84.2-94.8 | 57.14 | 41.0-72.3 | 2.11 | 1.5-3.0 | 0.17 | 0.09-0.30 | 66.1 | 56.7-74.6 | 86.6 | 75.5-94.0 | **73.1** |
| **APRI^4^** | 0.822 (0.758-0.875) | >0.7158 | 58.09 | 49.3-66.5 | 90.48 | 77.4-97.3 | 6.10 | 2.4-15.7 | 0.46 | 0.40-0.60 | 84.9 | 73.1-93.0 | 70.0 | 61.0-78.1 | **74.9** |
| **FIB4^1^** | 0.855 (0.795-0.903) | >1.2030 | 72.06 | 63.7-79.4 | 83.33 | 68.6-93.0 | 4.32 | 2.2-8.6 | 0.34 | 0.20-0.50 | 80.0 | 69.2-88.3 | 76.4 | 66.9-84.3 | **77.9** |
| **FIB4^2^** | 0.855 (0.795-0.903) | >1.3524 | 64.71 | 56.1-72.7 | 90.48 | 77.4-97.3 | 6.79 | 2.7-17.4 | 0.39 | 0.30-0.50 | 86.2 | 75.3-93.6 | 73.5 | 64.4-81.4 | **78.1** |
| **FIB4^3^** | 0.855 (0.795-0.903) | >0.8629 | 90.44 | 84.2-94.8 | 57.14 | 41.0-72.3 | 2.11 | 1.5-3.0 | 0.17 | 0.09-0.30 | 66.1 | 56.7-74.6 | 86.6 | 75.5-94.0 | **73.1** |
| **FIB4^4^** | 0.855 (0.795-0.903) | >1.3524 | 64.71 | 56.1-72.7 | 90.48 | 77.4-97.3 | 6.79 | 2.7-17.4 | 0.39 | 0.30-0.50 | 86.2 | 75.3-93.6 | 73.5 | 64.4-81.4 | **78.1** |
| **KING^1^** | 0.855 (0.795-0.903) | >10.6470 | 72.06 | 63.7-79.4 | 85.71 | 71.5-94.6 | 5.04 | 2.4-10.7 | 0.33 | 0.20-0.40 | 82.3 | 71.7-90.2 | 76.9 | 67.5-84.6 | **79.2** |
| **KING^2^** | 0.855 (0.795-0.903) | >10.6470 | 72.06 | 63.7-79.4 | 85.71 | 71.5-94.6 | 5.04 | 2.4-10.7 | 0.33 | 0.20-0.40 | 82.3 | 71.7-90.2 | 76.9 | 67.5-84.6 | **79.2** |
| **KING^3^** | 0.855 (0.795-0.903) | >6.9232 | 90.44 | 84.2-94.8 | 59.52 | 43.3-74.4 | 2.23 | 1.5-3.2 | 0.16 | 0.09-0.30 | 67.3 | 57.9-75.8 | 87.1 | 76.2-94.2 | **74.4** |
| **KING^4^** | 0.855 (0.795-0.903) | >12.8571 | 61.03 | 52.3-69.3 | 90.48 | 77.4-97.3 | 6.41 | 2.5-16.4 | 0.43 | 0.30-0.50 | 85.5 | 74.1-93.3 | 71.6 | 62.5-79.5 | **76.3** |
| **AAR^1^** | 0.554 (0.447-0.628) | >0.6486 | 54.01 | 45.3-62.6 | 61.90 | 45.6-76.4 | 1.42 | 0.9-2.1 | 0.74 | 0.60-1.00 | 56.7 | 45.2-67.7 | 59.3 | 48.9-69.2 | **58.1** |
| **AAR^2^** | 0.554 (0.447-0.628) | >0.6486 | 54.01 | 45.3-62.6 | 61.90 | 45.6-76.4 | 1.42 | 0.9-2.1 | 0.74 | 0.60-1.00 | 56.7 | 45.2-67.7 | 59.3 | 48.9-69.2 | **58.1** |
| **AAR^3^** | 0.554 (0.447-0.628) | >0.4538 | 90.51 | 84.3-94.9 | 11.90 | 4.0-25.6 | 1.03 | 0.9-1.2 | 0.80 | 0.30-2.10 | 48.7 | 40.7-56.7 | 57.6 | 33.2-79.5 | **49.6** |
| **AAR^4^** | 0.554 (0.447-0.628) | >1.0000 | 8.03 | 4.1-13.9 | 92.86 | 80.5-98.5 | 1.12 | 0.3-3.8 | 0.99 | 0.90-1.10 | 50.9 | 22.8-78.6 | 52.2 | 44.3-60.1 | **52.1** |
| **GUCI^1^** | 0.816 (0.751-0.870) | >30.4200 | 57.35 | 48.6-65.8 | 92.86 | 80.5-98.5 | 8.03 | 2.7-24.1 | 0.46 | 0.40-0.60 | 88.1 | 76.5-95.3 | 70.2 | 61.3-78.2 | **75.8** |
| **GUCI^2^** | 0.816 (0.751-0.870) | >30.4200 | 57.35 | 48.6-65.8 | 92.86 | 80.5-98.5 | 8.03 | 2.7-24.1 | 0.46 | 0.40-0.60 | 88.1 | 76.5-95.3 | 70.2 | 61.3-78.2 | **75.8** |
| **GUCI^3^** | 0.816 (0.751-0.870) | >15.5429 | 91.18 | 85.1-95.4 | 57.14 | 41.0-72.3 | 2.13 | 1.5-3.0 | 0.15 | 0.08-0.30 | 66.3 | 56.9-74.7 | 87.5 | 76.4-94.6 | **73.5** |
| **GUCI^4^** | 0.816 (0.751-0.870) | >30.1909 | 57.35 | 48.6-65.8 | 90.48 | 77.4-97.3 | 6.02 | 2.3-15.5 | 0.47 | 0.40-0.60 | 84.8 | 72.8-92.9 | 69.7 | 60.6-77.7 | **74.6** |
| **LOK^1^** | 0.683 (0.609-0.750) | >-1.5009 | 82.35 | 74.9-88.4 | 42.86 | 27.7-59.0 | 1.44 | 1.1-1.9 | 0.41 | 0.20-0.70 | 57.1 | 47.9-66.0 | 72.5 | 58.6-83.7 | **61.8** |
| **LOK^2^** | 0.683 (0.609-0.750) | >-0.8499 | 53.68 | 44.9-62.3 | 73.81 | 58.0-86.1 | 2.05 | 1.2-3.5 | 0.63 | 0.50-0.80 | 65.4 | 53.1-76.4 | 63.3 | 53.5-72.4 | **64.1** |
| **LOK^3^** | 0.683 (0.609-0.750) | >-1.6169 | 90.44 | 84.2-94.8 | 35.71 | 21.6-52.0 | 1.41 | 1.1-1.8 | 0.27 | 0.10-0.50 | 56.5 | 47.7-65.0 | 80.2 | 64.8-91.0 | **62.0** |
| **LOK^4^** | 0.683 (0.609-0.750) | >-0.4113 | 32.35 | 24.6-40.9 | 90.48 | 77.4-97.3 | 3.40 | 1.3-8.9 | 0.75 | 0.60-0.90 | 75.8 | 58.7-88.5 | 59.2 | 50.6-67.4 | **62.6** |
| **FORNS^1^** | 0.860 (0.799-0.908) | >4.5887 | 78.03 | 70.0-84.8 | 82.93 | 67.9-92.8 | 4.57 | 2.3-9.0 | 0.26 | 0.20-0.40 | 80.8 | 70.5-88.8 | 80.4 | 70.8-87.9 | **80.6** |
| **FORNS^2^** | 0.860 (0.799-0.908) | >5.0377 | 68.18 | 59.5-76.0 | 92.68 | 80.1-98.5 | 9.32 | 3.1-27.9 | 0.34 | 0.30-0.40 | 89.6 | 79.3-95.9 | 75.9 | 66.8-83.6 | **80.9** |
| **FORNS^3^** | 0.860 (0.799-0.908) | >3.6421 | 90.15 | 83.7-94.7 | 56.10 | 39.7-71.5 | 2.05 | 1.4-2.9 | 0.18 | 0.10-0.30 | 65.5 | 56.0-74.1 | 86.1 | 74.4-93.7 | **72.4** |
| **FORNS^4^** | 0.860 (0.799-0.908) | >4.9498 | 69.70 | 61.1-77.4 | 90.24 | 76.9-97.3 | 7.14 | 2.8-18.2 | 0.34 | 0.30-0.40 | 86.8 | 76.2-93.9 | 76.3 | 67.1-84.1 | **80.4** |
| **FI^1^** | 0.675 (0.598-0.745) | >1.8300 | 48.46 | 39.6-57.4 | 89.74 | 75.8-97.1 | 4.73 | 1.8-12.2 | 0.57 | 0.50-0.70 | 81.3 | 67.5-91.1 | 65.4 | 56.1-73.8 | **69.9** |
| **FI^2^** | 0.675 (0.598-0.745) | >1.8300 | 48.46 | 39.6-57.4 | 89.74 | 75.8-97.1 | 4.73 | 1.8-12.2 | 0.57 | 0.50-0.70 | 81.3 | 67.5-91.1 | 65.4 | 56.1-73.8 | **69.9** |
| **FI^3^** | 0.675 (0.598-0.745) | >0.9800 | 90.77 | 84.4-95.1 | 17.95 | 7.5-33.5 | 1.11 | 0.9-1.3 | 0.51 | 0.20-1.20 | 50.5 | 42.1-58.9 | 67.8 | 45.3-85.5 | **52.9** |
| **FI^4^** | 0.675 (0.598-0.745) | >2.1300 | 29.23 | 21.6-37.8 | 92.31 | 79.1-98.4 | 3.80 | 1.2-11.6 | 0.77 | 0.70-0.90 | 77.8 | 59.0-90.8 | 58.6 | 49.9-66.9 | **62.0** |
| **FCI^1^** | 0.522 (0.443-0.599) | >0.1943 | 20.00 | 13.5-27.9 | 94.74 | 82.3-99.4 | 3.80 | 0.9-15.3 | 0.84 | 0.80-0.90 | 77.8 | 53.9-93.0 | 56.2 | 47.8-64.4 | **58.9** |
| **FCI^2^** | 0.522 (0.443-0.599) | >0.1943 | 20.00 | 13.5-27.9 | 94.74 | 82.3-99.4 | 3.80 | 0.9-15.3 | 0.84 | 0.80-0.90 | 77.8 | 53.9-93.0 | 56.2 | 47.8-64.4 | **58.9** |
| **FCI^3^** | 0.522 (0.443-0.599) | >0.0386 | 90.00 | 83.5-94.6 | 15.79 | 6.0-31.3 | 1.07 | 0.9-1.2 | 0.63 | 0.30-1.60 | 49.7 | 41.3-58.0 | 63.1 | 39.6-82.8 | **51.4** |
| **FCI^4^** | 0.522 (0.443-0.599) | >0.1941 | 20.00 | 13.5-27.9 | 92.11 | 78.6-98.3 | 2.53 | 0.8-7.9 | 0.87 | 0.80-1.00 | 70.0 | 47.6-87.1 | 55.5 | 47.0-63.8 | **57.5** |
| **F>2** | | | | | | | | | | | | | | | |
|  | **AUC** | **Cutoff** | **Se(%)** | **CI** | **Sp(%)** | **CI** | **+LR(%)** | **CI** | **-LR(%)** | **CI** | **+PV(%)** | **CI** | **-PV(%)** | **CI** | **ACC(%)** |
| **AS^1^** | 0.920 (0.870-0.955) | >2.3967 | 81.43 | 70.3-89.7 | 91.67 | 84.8-96.1 | 9.77 | 5.2-18.4 | 0.20 | 0.10-0.30 | 77.4 | 63.1-88.2 | 93.4 | 87.6-97.0 | **89.0** |
| **AS^2^** | 0.920 (0.870-0.955) | >2.3967 | 81.43 | 70.3-89.7 | 91.67 | 84.8-96.1 | 9.77 | 5.2-18.4 | 0.20 | 0.10-0.30 | 77.4 | 63.1-88.2 | 93.4 | 87.6-97.0 | **89.0** |
| **AS^3^** | 0.920 (0.870-0.955) | >2.1267 | 90.00 | 80.5-95.9 | 69.44 | 59.8-77.9 | 2.95 | 2.2-4.0 | 0.14 | 0.07-0.30 | 50.9 | 39.5-62.1 | 95.2 | 88.8-98.5 | **74.8** |
| **AS^4^** | 0.920 (0.870-0.955) | >2.385 | 81.43 | 70.3-89.7 | 90.74 | 83.6-95.5 | 8.79 | 4.8-16.0 | 0.20 | 0.10-0.30 | 75.5 | 61.2-86.7 | 93.3 | 87.5-97.0 | **88.3** |
| **APRI^1^** | 0.882 (0.825-0.925) | >0.8069 | 80.00 | 68.7-88.6 | 86.11 | 78.1-92.0 | 5.76 | 3.5-9.3 | 0.23 | 0.10-0.40 | 66.9 | 52.9-79.0 | 92.5 | 86.2-96.5 | **84.5** |
| **APRI^2^** | 0.882 (0.825-0.925) | >1.0243 | 65.71 | 53.4-76.7 | 92.59 | 85.9-96.7 | 8.87 | 4.5-17.7 | 0.37 | 0.30-0.50 | 75.7 | 59.6-87.8 | 88.5 | 81.9-93.3 | **85.6** |
| **APRI^3^** | 0.882 (0.825-0.925) | >0.5769 | 90.00 | 80.5-95.9 | 62.04 | 52.2-71.2 | 2.37 | 1.8-3.1 | 0.16 | 0.08-0.30 | 45.4 | 35.0-56.2 | 94.6 | 87.6-98.3 | **69.3** |
| **APRI^4^** | 0.882 (0.825-0.925) | >0.9829 | 67.14 | 54.9-77.9 | 90.74 | 83.6-95.5 | 7.25 | 3.9-13.4 | 0.36 | 0.30-0.50 | 71.8 | 56.0-84.5 | 88.7 | 82.1-93.5 | **84.6** |
| **FIB4^1^** | 0.880 (0.823-0.924) | >1.7538 | 72.86 | 60.9-82.8 | 87.04 | 79.2-92.7 | 5.62 | 3.4-9.4 | 0.31 | 0.20-0.50 | 66.4 | 51.6-79.1 | 90.1 | 83.6-94.7 | **83.4** |
| **FIB4^2^** | 0.880 (0.823-0.924) | >2.0651 | 57.14 | 44.7-68.9 | 94.44 | 88.3-97.9 | 10.29 | 4.6-23.0 | 0.45 | 0.30-0.60 | 78.3 | 60.6-90.7 | 86.2 | 79.5-91.4 | **84.7** |
| **FIB4^3^** | 0.880 (0.823-0.924) | >1.2399 | 90.00 | 80.5-95.9 | 64.81 | 55.0-73.8 | 2.56 | 2.0-3.3 | 0.15 | 0.08-0.30 | 47.3 | 36.5-58.3 | 94.9 | 88.1-98.4 | **71.4** |
| **FIB4^4^** | 0.880 (0.823-0.924) | >1.8710 | 65.71 | 53.4-76.7 | 90.74 | 83.6-95.5 | 7.10 | 3.8-13.1 | 0.38 | 0.30-0.50 | 71.4 | 55.4-84.2 | 88.3 | 81.6-93.2 | **84.2** |
| **KING^1^** | 0.896 (0.842-0.937) | >12.8571 | 87.14 | 77.0-93.9 | 75.93 | 66.7-83.6 | 3.62 | 2.6-5.1 | 0.17 | 0.09-0.30 | 56.0 | 43.8-67.7 | 94.4 | 88.1-97.9 | **78.8** |
| **KING^2^** | 0.896 (0.842-0.937) | >18.0519 | 72.86 | 60.9-82.8 | 89.81 | 82.5-94.8 | 7.15 | 4.0-12.8 | 0.30 | 0.20-0.40 | 71.5 | 56.5-83.7 | 90.4 | 84.0-94.9 | **85.4** |
| **KING^3^** | 0.896 (0.842-0.937) | >12.0313 | 90.00 | 80.5-95.9 | 71.3 | 61.8-79.6 | 3.14 | 2.3-4.3 | 0.14 | 0.07-0.30 | 52.4 | 40.9-63.8 | 95.3 | 89.0-98.5 | **76.2** |
| **KING^4^** | 0.896 (0.842-0.937) | >20.4209 | 62.86 | 50.5-74.1 | 90.74 | 83.6-95.5 | 6.79 | 3.7-12.6 | 0.41 | 0.30-0.60 | 70.5 | 54.2-83.7 | 87.4 | 80.7-92.5 | **83.5** |
| **AAR^1^** | 0.617 (0.542-0.689) | >0.7363 | 45.07 | 33.2-57.3 | 77.78 | 68.8-85.2 | 2.03 | 1.3-3.1 | 0.71 | 0.60-0.90 | 41.6 | 27.8-56.4 | 80.1 | 72.1-86.6 | **69.3** |
| **AAR^2^** | 0.617 (0.542-0.689) | >1.3846 | 1.41 | 0.1-7.6 | 100.00 | 96.6-100 | - | - | 0.99 | 1.00-1.00 | 100.0 | 50.0-100.0 | 74.3 | 67.2-80.5 | **74.4** |
| **AAR^3^** | 0.617 (0.542-0.689) | >0.4538 | 90.14 | 80.7-95.9 | 10.19 | 5.2-17.5 | 1.00 | 0.9-1.1 | 0.97 | 0.40-2.40 | 26.1 | 19.5-33.6 | 74.6 | 49.0-91.8 | **31.0** |
| **AAR^4^** | 0.617 (0.542-0.689) | >0.9118 | 15.49 | 8.0-26.0 | 90.74 | 83.6-95.5 | 1.67 | 0.8-3.7 | 0.93 | 0.80-1.00 | 37.0 | 16.4-61.8 | 75.3 | 67.9-81.8 | **71.2** |
| **GUCI^1^** | 0.881 (0.825-0.925) | >32.1370 | 82.86 | 72.0-90.8 | 82.41 | 73.9-89.1 | 4.71 | 3.1-7.2 | 0.21 | 0.10-0.40 | 62.3 | 49.0-74.4 | 93.2 | 87.0-97.0 | **82.5** |
| **GUCI^2^** | 0.881 (0.825-0.925) | >48.9583 | 58.57 | 46.2-70.2 | 95.37 | 89.5-98.5 | 12.65 | 5.3-30.5 | 0.43 | 0.30-0.60 | 81.6 | 64.3-92.9 | 86.8 | 80.1-91.8 | **85.8** |
| **GUCI^3^** | 0.881 (0.825-0.925) | >26.0556 | 90.00 | 80.5-95.9 | 67.59 | 57.9-76.3 | 2.78 | 2.1-3.7 | 0.15 | 0.07-0.30 | 49.4 | 38.3-60.5 | 95.1 | 88.5-98.5 | **73.4** |
| **GUCI^4^** | 0.881 (0.825-0.925) | >38.5299 | 71.43 | 59.4-81.6 | 90.74 | 83.6-95.5 | 7.71 | 4.2-14.2 | 0.31 | 0.20-0.50 | 73.0 | 57.7-85.2 | 90.0 | 83.6-94.6 | **85.7** |
| **LOK^1^** | 0.771 (0.702-0.831) | >-0.7447 | 70.00 | 57.9-80.4 | 75.00 | 65.7-82.8 | 2.80 | 2.0-4.0 | 0.40 | 0.30-0.60 | 49.6 | 36.9-62.3 | 87.7 | 80.1-93.1 | **73.7** |
| **LOK^2^** | 0.771 (0.702-0.831) | >-0.1938 | 34.29 | 23.3-46.6 | 93.52 | 87.1-97.4 | 5.29 | 2.4-11.6 | 0.70 | 0.60-0.80 | 65.0 | 43.0-83.1 | 80.2 | 73.0-86.2 | **78.1** |
| **LOK^3^** | 0.771 (0.702-0.831) | >-1.3542 | 90.00 | 80.5-95.9 | 37.04 | 27.9-46.9 | 1.43 | 1.2-1.7 | 0.27 | 0.10-0.60 | 33.4 | 25.2-42.5 | 91.3 | 80.3-97.3 | **50.8** |
| **LOK^4^** | 0.771 (0.702-0.831) | >-0.2708 | 37.14 | 25.9-49.5 | 90.74 | 83.6-95.5 | 4.01 | 2.1-7.8 | 0.69 | 0.60-0.80 | 58.5 | 38.8-76.4 | 80.4 | 73.1-86.5 | **76.8** |
| **FORNS^1^** | 0.857 (0.796-0.905) | >5.0262 | 91.18 | 81.8-96.7 | 68.57 | 58.8-77.3 | 2.90 | 2.2-3.9 | 0.13 | 0.06-0.30 | 50.5 | 39.1-61.8 | 95.7 | 89.2-98.8 | **74.4** |
| **FORNS^2^** | 0.857 (0.796-0.905) | >6.4581 | 52.94 | 40.4-65.2 | 93.33 | 86.7-97.3 | 7.94 | 3.8-16.8 | 0.50 | 0.40-0.70 | 73.6 | 55.1-87.5 | 85.0 | 77.9-90.4 | **82.8** |
| **FORNS^3^** | 0.857 (0.796-0.905) | >5.0262 | 91.18 | 81.8-96.7 | 68.57 | 58.8-77.3 | 2.90 | 2.2-3.9 | 0.13 | 0.06-0.30 | 50.5 | 39.1-61.8 | 95.7 | 89.2-98.8 | **74.4** |
| **FORNS^4^** | 0.857 (0.796-0.905) | >6.2584 | 57.35 | 44.8-69.3 | 90.48 | 83.2-95.3 | 6.02 | 3.2-11.2 | 0.47 | 0.40-0.60 | 67.9 | 50.6-82.3 | 85.8 | 78.7-91.2 | **81.9** |
| **FI^1^** | 0.764 (0.693-0.826) | >1.8300 | 70.77 | 58.2-81.4 | 79.81 | 70.8-87.0 | 3.50 | 2.3-5.3 | 0.37 | 0.20-0.50 | 55.2 | 41.3-68.5 | 88.6 | 81.2-93.8 | **77.5** |
| **FI^2^** | 0.764 (0.693-0.826) | >2.2400 | 41.54 | 29.4-54.4 | 93.27 | 86.6-97.3 | 6.17 | 2.9-13.3 | 0.63 | 0.50-0.80 | 68.4 | 47.4-85.1 | 82.0 | 74.6-87.9 | **79.8** |
| **FI^3^** | 0.764 (0.693-0.826) | >1.2500 | 89.23 | 79.1-95.6 | 34.62 | 25.6-44.6 | 1.36 | 1.2-1.6 | 0.31 | 0.10-0.70 | 32.4 | 24.2-41.6 | 90.1 | 78.1-96.9 | **48.8** |
| **FI^4^** | 0.764 (0.693-0.826) | >2.1600 | 44.62 | 32.3-57.5 | 90.38 | 83.0-95.3 | 4.64 | 2.4-8.9 | 0.61 | 0.50-0.80 | 62.0 | 42.9-78.7 | 82.3 | 74.8-88.3 | **78.5** |
| **FCI^1^** | 0.696 (0.621-0.765) | >0.1513 | 47.69 | 35.1-60.5 | 84.47 | 76.0-90.9 | 3.07 | 1.8-5.2 | 0.62 | 0.50-0.80 | 51.9 | 35.6-67.9 | 82.1 | 74.3-88.4 | **74.9** |
| **FCI^2^** | 0.696 (0.621-0.765) | >0.2353 | 26.15 | 16.0-38.5 | 98.06 | 93.2-99.8 | 13.47 | 3.2-56.4 | 0.75 | 0.60-0.90 | 82.6 | 52.2-97.4 | 79.1 | 71.8-85.2 | **79.4** |
| **FCI^3^** | 0.696 (0.621-0.765) | >0.0459 | 90.77 | 81.0-96.5 | 25.24 | 17.2-34.8 | 1.21 | 1.1-1.4 | 0.37 | 0.20-0.80 | 29.9 | 22.2-38.5 | 88.6 | 73.3-96.8 | **42.3** |
| **FCI^4^** | 0.696 (0.621-0.765) | >0.1804 | 33.85 | 22.6-46.6 | 90.29 | 82.9-95.2 | 3.49 | 1.8-6.9 | 0.73 | 0.60-0.90 | 55.1 | 34.5-74.4 | 79.5 | 71.9-85.9 | **75.6** |
| **F>3** | | | | | | | | | | | | | | | |
|  | **AUC** | **Cutoff** | **Se(%)** | **CI** | **Sp(%)** | **CI** | **+LR(%)** | **CI** | **-LR(%)** | **CI** | **+PV(%)** | **CI** | **-PV(%)** | **CI** | **ACC(%)** |
| **AS^1^** | 0.923 (0.873-0.957) | >2.5393 | 96.77 | 83.3-99.9 | 78.91 | 71.4-85.2 | 4.59 | 3.3-6.3 | 0.04 | 0.01-0.30 | 38.5 | 25.5-52.9 | 99.4 | 96.0-100.0 | **81.1** |
| **AS^2^** | 0.923 (0.873-0.957) | >3.0687 | 67.74 | 48.6-83.3 | 93.88 | 88.7-97.2 | 11.06 | 5.6-21.8 | 0.34 | 0.20-0.60 | 60.1 | 38.3-79.3 | 95.5 | 90.9-98.2 | **90.7** |
| **AS^3^** | 0.923 (0.873-0.957) | >2.6063 | 90.32 | 74.2-98.0 | 82.31 | 75.2-88.1 | 5.11 | 4.5-5.9 | 0.12 | 0.04-0.40 | 41.1 | 26.8-56.5 | 98.4 | 94.5-99.8 | **83.3** |
| **AS^4^** | 0.923 (0.873-0.957) | >2.9035 | 74.19 | 55.4-88.1 | 90.48 | 84.5-94.7 | 7.79 | 4.5-13.4 | 0.29 | 0.20-0.50 | 51.5 | 32.7-70.1 | 96.3 | 91.8-98.7 | **88.5** |
| **APRI^1^** | 0.887 (0.831-0.930) | >0.7401 | 100.00 | 88.8-100. | 67.35 | 59.1-74.8 | 3.06 | 2.4-3.9 | 0 | - | 29.5 | 19.3-41.4 | 100.0 | 96.5-100.0 | **71.3** |
| **APRI^2^** | 0.887 (0.831-0.930) | >2.0504 | 35.48 | 19.2-54.6 | 97.96 | 94.2-99.6 | 17.39 | 5.2-58.7 | 0.66 | 0.50-0.90 | 70.3 | 35.1-93.5 | 91.8 | 86.5-95.4 | **90.5** |
| **APRI^3^** | 0.887 (0.831-0.930) | >0.8386 | 90.32 | 74.2-98.0 | 73.47 | 65.6-80.4 | 3.40 | 2.5-4.6 | 0.13 | 0.04-0.40 | 31.7 | 20.3-45.0 | 98.2 | 93.9-99.8 | **75.5** |
| **APRI^4^** | 0.887 (0.831-0.930) | >1.4469 | 51.61 | 33.1-69.8 | 90.48 | 84.5-94.7 | 5.42 | 3.0-9.9 | 0.53 | 0.40-0.80 | 42.5 | 23.2-63.7 | 93.2 | 88.0-96.6 | **85.8** |
| **FIB4^1^** | 0.858 (0.978-0.906) | >1.5558 | 93.55 | 78.6-99.2 | 70.07 | 62.0-77.3 | 3.13 | 2.4-4.1 | 0.09 | 0.02-0.40 | 29.9 | 19.2-42.4 | 98.8 | 94.5-99.9 | **72.9** |
| **FIB4^2^** | 0.858 (0.978-0.906) | >3.4289 | 29.03 | 14.2-48.0 | 97.28 | 93.2-99.3 | 10.67 | 3.5-32.4 | 0.73 | 0.60-0.90 | 59.3 | 25.7-87.4 | 91.0 | 85.5-94.8 | **89.1** |
| **FIB4^3^** | 0.858 (0.978-0.906) | >1.5887 | 90.32 | 74.2-98.0 | 70.75 | 62.7-78.0 | 3.09 | 2.3-4.1 | 0.14 | 0.05-0.40 | 29.6 | 18.9-42.2 | 98.2 | 93.6-99.8 | **73.1** |
| **FIB4^4^** | 0.858 (0.978-0.906) | >2.4433 | 58.06 | 39.1-75.5 | 90.48 | 84.5-94.7 | 6.10 | 3.4-10.9 | 0.46 | 0.30-0.70 | 45.4 | 26.3-65.6 | 94.1 | 89.0-97.3 | **86.6** |
| **KING^1^** | 0.878 (0.821-0.923) | >16.6327 | 90.32 | 74.2-98.0 | 73.47 | 65.6-80.4 | 3.40 | 2.5-4.6 | 0.13 | 0.04-0.40 | 31.7 | 20.3-45.0 | 98.2 | 93.9-99.8 | **75.5** |
| **KING^2^** | 0.878 (0.821-0.923) | >50.6849 | 29.03 | 14.2-48.0 | 98.64 | 95.2-99.8 | 21.34 | 4.8-94.0 | 0.72 | 0.60-0.90 | 74.4 | 34.4-96.6 | 91.1 | 85.7-94.9 | **90.3** |
| **KING^3^** | 0.878 (0.821-0.923) | >16.6327 | 90.32 | 74.2-98.0 | 73.47 | 65.6-80.4 | 3.40 | 2.5-4.6 | 0.13 | 0.04-0.40 | 31.7 | 20.3-45.0 | 98.2 | 93.9-99.8 | **75.5** |
| **KING^4^** | 0.878 (0.821-0.923) | >29.2827 | 51.61 | 33.1-69.8 | 90.48 | 84.5-94.7 | 5.42 | 3.0-9.9 | 0.53 | 0.40-0.80 | 42.5 | 23.2-63.7 | 93.2 | 88.0-96.6 | **85.8** |
| **AAR^1^** | 0.643 (0.568-0.713) | >0.5444 | 96.77 | 83.3-99.9 | 31.08 | 23.7-39.2 | 1.40 | 1.2-1.6 | 0.10 | 0.01-0.70 | 16.1 | 10.2-23.6 | 98.6 | 90.2-100.0 | **39.0** |
| **AAR^2^** | 0.643 (0.568-0.713) | >1.3846 | 3.23 | 0.08-16.7 | 100.00 | 97.5-100.0 | - | - | 0.97 | 0.90-1.00 | 100 | 50.0-100.0 | 88.3 | 82.7-92.7 | **88.4** |
| **AAR^3^** | 0.643 (0.568-0.713) | >0.5490 | 90.32 | 74.2-98.0 | 31.08 | 23.7-39.2 | 1.31 | 1.1-1.5 | 0.31 | 0.10-0.90 | 15.2 | 9.4-22.6 | 95.9 | 86.3-99.5 | **38.2** |
| **AAR^4^** | 0.643 (0.568-0.713) | >0.9600 | 16.13 | 5.5-33.7 | 90.54 | 84.6-94.7 | 1.71 | 0.7-4.4 | 0.93 | 0.80-1.10 | 18.9 | 4.6-43.9 | 88.8 | 82.8-93.2 | **81.6** |
| **GUCI^1^** | 0.896 (0.841-0.936) | >32.9412 | 100.00 | 88.8-100.0 | 70.07 | 62.0-77.3 | 3.34 | 2.6-4.3 | 0 | - | 31.3 | 20.6-43.7 | 100.00 | 96.7-100.0 | **73.7** |
| **GUCI^2^** | 0.896 (0.841-0.936) | >100.7660 | 35.48 | 19.2-54.6 | 98.64 | 95.2-99.8 | 26.08 | 6.1-111.9 | 0.65 | 0.50-0.80 | 78.1 | 40.3-97.3 | 91.8 | 86.6-95.5 | **91.1** |
| **GUCI^3^** | 0.896 (0.841-0.936) | >35.6000 | 90.32 | 74.2-98.0 | 73.47 | 65.6-80.4 | 3.40 | 2.5-4.6 | 0.13 | 0.04-0.40 | 31.7 | 20.3-45.0 | 98.2 | 93.9-99.8 | **75.5** |
| **GUCI^4^** | 0.896 (0.841-0.936) | >63.1628 | 54.84 | 36.0-72.7 | 90.48 | 84.5-94.7 | 5.76 | 3.2-10.4 | 0.50 | 0.30-0.70 | 44.0 | 24.7-64.6 | 93.6 | 88.5-97.0 | **86.2** |
| **LOK^1^** | 0.882 (0.825-0.926) | >-0.3791 | 83.87 | 66.3-94.5 | 85.71 | 79.0-90.9 | 5.87 | 3.8-9.0 | 0.19 | 0.08-0.40 | 44.5 | 28.8-61.0 | 97.5 | 93.3-99.4 | **85.5** |
| **LOK^2^** | 0.882 (0.825-0.926) | >0.7077 | 16.13 | 5.5-33.7 | 99.32 | 96.3-100. | 23.71 | 2.9-195.9 | 0.84 | 0.70-1.00 | 76.4 | 20.3-99.5 | 89.7 | 84.1-93.8 | **89.3** |
| **LOK^3^** | 0.882 (0.825-0.926) | >-0.6845 | 90.32 | 74.2-98.0 | 73.47 | 65.6-80.4 | 3.40 | 2.5-4.6 | 0.13 | 0.04-0.40 | 31.7 | 20.3-45.0 | 98.2 | 93.9-99.8 | **75.5** |
| **LOK^4^** | 0.882 (0.825-0.926) | >-0.2643 | 67.74 | 48.6-83.3 | 90.48 | 84.5-94.7 | 7.11 | 4.1-12.4 | 0.36 | 0.20-0.60 | 49.2 | 30.3-68.3 | 95.4 | 90.6-98.1 | **87.7** |
| **FORNS^1^** | 0.849 (0.787-0.899) | >6.5692 | 70.00 | 50.6-85.3 | 85.31 | 78.4-90.7 | 4.77 | 3.0-7.5 | 0.35 | 0.20-0.60 | 39.4 | 23.6-57.0 | 95.4 | 90.4-98.3 | **83.5** |
| **FORNS^2^** | 0.849 (0.787-0.899) | >7.0365 | 43.33 | 25.5-62.6 | 95.8 | 91.1-98.4 | 10.33 | 4.3-25.0 | 0.59 | 0.40-0.80 | 58.5 | 31.0-82.6 | 92.5 | 87.2-96.1 | **89.5** |
| **FORNS^3^** | 0.849 (0.787-0.899) | >5.0706 | 90.00 | 73.5-97.9 | 55.94 | 47.4-64.2 | 2.04 | 1.6-2.5 | 0.18 | 0.06-0.50 | 21.8 | 13.6-32.1 | 97.6 | 91.8-99.7 | **60.0** |
| **FORNS^4^** | 0.849 (0.787-0.899) | >6.8498 | 53.33 | 34.3-71.7 | 90.21 | 84.1-94.5 | 5.45 | 3.0-9.9 | 0.52 | 0.40-0.80 | 42.6 | 23.3-63.8 | 93.4 | 88.1-96.8 | **85.8** |
| **FI^1^** | 0.805 (0.737-0.861) | >2.0300 | 75.86 | 56.5-89.7 | 79.29 | 71.6-85.7 | 3.66 | 2.5-5.4 | 0.30 | 0.20-0.60 | 33.3 | 20.1-48.7 | 96.0 | 90.8-98.7 | **78.9** |
| **FI^2^** | 0.805 (0.737-0.861) | >3.1200 | 10.34 | 2.2-27.4 | 99.29 | 96.1-100. | 14.48 | 1.6-134.4 | 0.90 | 0.80-1.00 | 66.4 | 9.3-99.1 | 89.0 | 83.2-93.4 | **88.6** |
| **FI^3^** | 0.805 (0.737-0.861) | >1.4200 | 93.10 | 77.2-99.2 | 41.43 | 33.2-50.1 | 1.59 | 1.3-1.9 | 0.17 | 0.04-0.60 | 17.8 | 11.0-26.5 | 97.8 | 90.5-99.9 | **47.6** |
| **FI^4^** | 0.805 (0.737-0.861) | >2.3600 | 48.28 | 29.4-67.5 | 90.00 | 83.8-94.4 | 4.83 | 2.6-9.0 | 0.57 | 0.40-0.80 | 39.7 | 20.5-61.5 | 92.7 | 87.2-96.4 | **85.0** |
| **FCI^1^** | 0.750 (0.677-0.813) | >0.2069 | 48.28 | 29.4-67.5 | 92.81 | 87.2-96.5 | 6.71 | 3.3-13.6 | 0.56 | 0.40-0.80 | 47.8 | 25.3-70.9 | 92.9 | 87.5-96.5 | **87.5** |
| **FCI^2^** | 0.750 (0.677-0.813) | >0.3324 | 31.03 | 15.3-50.8 | 100.00 | 97.4-100.0 | - | - | 0.69 | 0.50-0.90 | 100.00 | 54.1-100.0 | 91.4 | 86.0-95.2 | **91.7** |
| **FCI^3^** | 0.750 (0.677-0.813) | >0.0550 | 93.10 | 77.2-99.2 | 27.34 | 20.1-35.5 | 1.28 | 1.1-1.5 | 0.25 | 0.06-1.00 | 14.9 | 9.2-22.3 | 96.7 | 85.8-99.8 | **35.2** |
| **FCI^4^** | 0.750 (0.677-0.813) | >0.1974 | 48.28 | 29.4-67.5 | 90.65 | 84.5-94.9 | 5.16 | 2.7-9.8 | 0.57 | 0.40-0.80 | 41.3 | 21.4-63.5 | 92.8 | 87.3-96.4 | **85.6** |

^1^Youden index criterion; ^2^Optimal criterion;^3^90% Sensitivity criterion; ^4^90% Specificity criterion; Se, Sensitivity; Sp, Specificity; +LR, Positive Likelihood ratio; -LR, Negative Likelihood ratio; +PV, Positive predictive value; -PV, Negative predictive value; ACC, Accuracy.
